# Machine Learning Models to Evaluate County-Level Incidence of Diagnosed Diabetes and Sociodemographic Factors

**DOI:** 10.1016/j.ajmo.2026.100132

**Published:** 2026-03-27

**Authors:** Alexander S. Keigley, Shant Ayanian, Sagar B. Dugani

**Affiliations:** a Admission and Transfer Center, Mayo Clinic, Rochester, MN; b Division of Hospital Internal Medicine, Mayo Clinic, Rochester, MN; c Division of Health Care Delivery Research, Robert D. and Patricia E. Kern Center for the Science of Health Care Delivery, Mayo Clinic, Rochester, MN

**Keywords:** Diabetes, Machine learning, Social determinants of health, Social vulnerability index

## Abstract

**Aims:**

To evaluate county-level incidence of diagnosed diabetes and key sociodemographic factors in a high-dimensional, nonlinear setting.

**Methods:**

This temporally aggregated observational study used US Centers for Disease Control and Prevention data on county-level incidence of diagnosed diabetes, from 2004 to 2019, and 34 sociodemographic factors from public databases. We defined counties as *higher-burden* if diabetes incidence was >12.6 per 1000 persons (1 standard deviation [SD] above sample mean). As relationships between sociodemographic factors and diabetes incidence may be nonlinear and involve complex interactions, we trained three machine learning models to estimate incidence (elastic net regression), classify counties as higher-burden (eXtreme Gradient Boosting [XGBoost], support vector machine [SVM]), and identify feature importance. Model performance was evaluated using fivefold cross-validation, with stratified folds for XGBoost and SVM models.

**Results:**

Overall, 500 of 3114 counties (16.1%) were of higher-burden. Elastic net regression showed good predictive performance for estimating diabetes incidence (*R*^2^ 0.78 [95% CI, 0.75-0.80]). For classification of higher-burden counties, SVM and XGBoost showed high discrimination with AUROC of 0.962 (95% CI, 0.948-0.974) and 0.957 (95% CI, 0.941-0.971), respectively. Sensitivity analyses using alternative definitions of higher-burden counties (mean + 0.75 × SD; mean + 1.25 × SD) yielded comparable results. Across all three models, key county-level features contributing to model predictions were percentages of *children living with grandparent householders* and of people with*Limited English*.

**Conclusions:**

Machine learning models demonstrated consistent performance in estimating and classifying county-level diabetes incidence, with high discrimination for identifying higher-burden counties. Sociodemographic factors, including *children living with grandparent householders*, may inform tailored public health interventions.

## Introduction

In 2021, an estimated 38 million US adults aged 18 years or older had diabetes, a burden influenced by both individual- and population-level factors.^1^^,^^2^ While many studies have examined the social determinants of diabetes incidence using survey data such as Behavioral Risk Factor Surveillance System (BRFSS) and National Health and Nutrition Examination Survey (NHANES), few have comprehensively modeled these factors using machine learning (ML) approaches applied to county-level, population-based data.

Prior studies have primarily focused on individual-level factors associated with incident diabetes, including lifestyle behaviors, metabolic conditions, and biomarkers.^3^, ^4^, ^5^, ^6^, ^7^, ^8^ Recent studies have also highlighted the importance of population-level factors.^2^^,^^9^, ^10^, ^11^ We used US Centers for Disease Control and Prevention (CDC) data and reported an increase in county-level incidence of diagnosed diabetes with increasing rurality.^2^ In a US study, counties with a lower incidence of diagnosed diabetes had more exercise opportunities, better access to healthy food, and lower unemployment, among other factors, with 42% of diabetes variation across counties attributed to socioeconomic and health-related characteristics.^9^ In support, other studies reported the inverse association between neighborhood greenspace and risk of diabetes.^12^, ^13^, ^14^, ^15^ However, most studies have evaluated a limited number of sociodemographic factors and relied on classical regression approaches, which may not fully capture nonlinear relationships and interactions among county-level factors. In this context, ML approaches offer advantages by accommodating high-dimensional data and modeling complex, nonlinear associations. These approaches may help identify modifiable factors to inform diabetes prevention efforts.

To address this knowledge gap, we used US CDC data on county-level incidence of diagnosed diabetes, from 2004 to 2019, among adults aged 20 years or older. We reviewed county-level data on more than 600 sociodemographic factors and applied three ML approaches to evaluate sociodemographic factors and incidence of diagnosed diabetes. We hypothesized that ML models would capture nonlinear associations and identify sociodemographic factors that predict county-level diabetes incidence.

## Material and Methods

The study was reported using STROBE guidelines. The use of public data was deemed “not human subjects” research by the Mayo Clinic institutional review board.

### Data Source

We obtained US CDC data on county-level incidence of diagnosed diabetes, as described.^2^^,^^16^ Briefly, BRFSS is a state-based telephone survey, in which adults aged 20 years or older self-reported medical conditions, including diabetes (“Has a doctor ever told you that you have diabetes?”). Incidence of diagnosed diabetes was based on diagnosis within a year prior to the survey, combined with reported age at the survey and age at diabetes diagnosis.^2^^,^^16^ The CDC used BRFSS survey data, US Census Bureau population estimates, and small area estimation to generate county-level incidence rates of diagnosed diabetes. From the CDC portal, we extracted data on incidence rates, available for counties and county equivalents, annually from 2004 to 2019, which includes the latest available data. Given the objective to characterize patterns in incidence rather than temporal trends, we used the median value of diabetes incidence to improve the stability of estimates, particularly for smaller counties.

### County-level Sociodemographic Factors Across Multiple Domains

We extracted data on county-level sociodemographic factors across multiple domains. All predictors were county-level measures derived from publicly available datasets and do not represent individual-level electronic health record data. The CDC county-level social vulnerability index (SVI) is comprised of four themes (socioeconomic status, household characteristics, racial, and ethnic minority status, and housing type and transportation), which were derived using 16 factors: persons below 150% poverty, persons unemployed, housing cost-burden, persons with no high school diploma, persons with no health insurance, persons aged 65 and older, persons aged 17 and younger, civilians with a disability, single-parent households, persons with English language proficiency, persons with minority status, multi-unit structures, mobile homes, crowding, no vehicle, and group quarters, from the year 2020.^17^ Previous reported years of SVI (ie, prior to 2020) included two factors—per capita income and poverty status—which were included for analysis.^17^ We reviewed approximately 600 county-level sociodemographic factors in the Agency for Healthcare Research and Quality (AHRQ) database and selected six factors that were minimally correlated with the SVI factors (selection is described in the following section).^18^ We included 10 additional factors that were not reported in other databases: sex ratio, college education, food environment index, primary care physicians, other primary care professional, inactivity, obesity, adult population, region, and county rurality.^16^^,^^19^^,^^20^ County-level rurality was based on the 2013 National Center for Health Statistics/CDC categories: large central metropolitan (most urban), large fringe metropolitan, medium metropolitan, small metropolitan, micropolitan (large rural), and noncore (most rural), as described in Supplementary Table 1.^19^ US regions were based on US Census Bureau classification, as described in Supplementary Table 2.^21^ During data assembly, when a county-level predictor was available in multiple years but missing for intermediate years, we used linear interpolation within county to impute intermediate values, as described.^2^ The 34 factors were aggregated into a single dataset which contained the median annual values per county, and are further defined in Supplementary Table 3.

**Table 1:** Sociodemographic Factors by County-Level Burden of Incidence of Diagnosed Diabetes

	All Counties (*N* = 3114)	Higher-Burden Counties (*N* = 500)	Lower-Burden Counties (*N* = 2614)	*P* Value
Incidence of diagnosed diabetes, per 1000 persons	10.3 ± 2.3	14.0 ± 1.2	9.6 ± 1.7	<.0001
Adult population	72,940 ± 232,991	22,880 ± 23,215	82,516 ± 252,979	.21
Over 65 y old	18.36 ± 4.54	18.22 ± 3.36	18.38 ± 4.73	.88
Under 17 y old	22.37 ± 3.38	22.51 ± 3.27	22.35 ± 3.41	.85
Sex ratio	1.00 ± 0.11	0.98 ± 0.11	1.01 ± 0.10	.27
No high school diploma	13.42 ± 6.23	19.11 ± 4.33	12.34 ± 5.94	<.0001
Some college	55.68 ± 11.41	45.84 ± 7.80	57.56 ± 11.02	<.0001
Per capita income	25,017 ± 5907	19,823 ± 3177	26,011 ± 5787	<.0001
Unemployment rate	22.09 ± 5.00	23.08 ± 4.94	21.90 ± 4.99	.36
Below poverty line	5.94 ± 2.68	8.80 ± 3.15	5.40 ± 2.20	<.0001
Below 150% of poverty line	24.25 ± 8.29	33.70 ± 7.58	22.44 ± 7.11	<.0001
Uninsured	9.52 ± 4.99	11.45 ± 5.06	9.15 ± 4.89	.08
Food environment index	7.17 ± 1.21	6.09 ± 1.34	7.37 ± 1.06	<.001
Veterans	10.03 ± 2.60	9.10 ± 2.17	10.21 ± 2.64	.08
Minority population	23.46 ± 20.04	37.14 ± 22.68	20.85 ± 18.38	.003
Disabled	15.91 ± 4.26	19.60 ± 4.04	15.20 ± 3.92	<.0001
Limited English	1.68 ± 2.80	0.76 ± 1.11	1.86 ± 2.98	.07
Single-parent households	7.91 ± 2.56	9.70 ± 3.00	7.56 ± 2.31	.003
Children living with grandparent householders	8.02 ± 4.27	13.49 ± 4.19	6.98 ± 3.40	<.0001
Mobile homes	12.81 ± 9.40	22.32 ± 9.19	10.99 ± 8.27	<.0001
High-density housing	4.67 ± 5.70	2.10 ± 2.09	5.17 ± 6.03	.01
Crowded housing	2.34 ± 2.09	2.56 ± 1.85	2.29 ± 2.13	.60
Group quarters	3.51 ± 4.48	3.87 ± 4.79	3.44 ± 4.42	.72
Housing cost-burden	16.36 ± 6.41	23.92 ± 6.43	14.91 ± 5.30	<.0001
No vehicle available	6.30 ± 4.13	8.45 ± 3.01	5.88 ± 4.18	.009
No computing device	15.61 ± 6.56	22.78 ± 6.54	14.24 ± 5.60	<.0001
Smartphone	70.23 ± 8.24	63.98 ± 7.72	71.43 ± 7.79	<.001
Any broadband access	74.03 ± 8.97	64.22 ± 8.58	75.90 ± 7.74	<.0001
No kitchen	4.64 ± 3.32	5.82 ± 2.69	4.42 ± 3.38	.08
Primary care physicians	52.37 ± 35.54	39.50 ± 24.01	54.83 ± 36.83	.06
Other primary care professionals	67.75 ± 51.38	64.05 ± 40.94	68.46 ± 53.13	.72
Obesity	25.12 ± 3.46	27.16 ± 3.93	24.73 ± 3.22	<.0001
Inactivity	21.42 ± 3.39	24.81 ± 3.64	20.78 ± 2.92	<.0001
County-level rurality				<.001
Large central metro	67 (2.2%)	0 (0.0%)	67 (2.6%)	
Large fringe metro	366 (11.8%)	27 (5.4%)	339 (13.0%)	
Medium metro	368 (11.8%)	45 (9.0%)	323 (12.4%)	
Small metro	357 (11.5%)	53 (10.6%)	304 (11.6%)	
Micropolitan	636 (20.4%)	105 (21.0%)	531 (20.3%)	
Noncore	1320 (42.4%)	270 (54.0%)	1050 (40.2%)	
Region				<.0001
Midwest	1050 (33.7%)	17 (3.4%)	1033 (39.5%)	
Northeast	209 (6.7%)	2 (0.4%)	207 (7.9%)	
South	1420 (45.6%)	476 (95.2%)	944 (36.1%)	
West	435 (14.0%)	5 (1.0%)	430 (16.4%)	

Data reported as frequency (%) or mean (±standard deviation). Counties were categorized based on the CDC-reported incidence of diagnosed diabetes per 1000 persons: higher-burden (>12.6) and lower-burden (≤12.6). Statistical significance was based on Wilcoxon rank-sum (continuous factors) and chi-square (categorical factors) tests.

**Table 2:** Machine Learning Metrics for County-Level Incidence of Diagnosed Diabetes

Regression Performance Metrics (95% Confidence Interval)					
Model	MAE	RMSE	*R*^2^		
Elastic Net Regression	0.810 (0.767-0.852)	1.057 (0.994-1.116)	0.778 (0.747-0.804)		
Classification Performance Metrics (95% Confidence Interval)					
	Accuracy	Precision	Recall	F1 Score	AUROC
SVM	0.924 (0.906-0.940)	0.821 (0.748-0.885)	0.673 (0.596-0.748)	0.740 (0.675-0.796)	0.962 (0.948-0.974)
XGBoost	0.923 (0.905-0.939)	0.800 (0.728-0.864)	0.693 (0.617-0.762)	0.743 (0.678-0.793)	0.957 (0.941-0.971)

Model performance for county-level incidence of diagnosed diabetes. Elastic net regression predicted county-level incidence of diagnosed diabetes, whereas SVM and XGBoost modeled confidence in classifying counties as having a higher-burden (>12.6 per 1000 persons) or lower-burden (≤12.6 per 1000 persons) of incidence of diagnosed diabetes. The 95% confidence intervals were estimated using nonparametric bootstrap resampling of the test set (2000 resamples; stratified bootstrap for classification).

AUROC = area under receiver operating characteristic; MAE = mean absolute error; RMSE = root mean squared error; SVM = support vector machine; XGBoost = eXtreme Gradient Boosting.

### Feature Selection

To reduce redundancy and multicollinearity, we screened predictors for high pairwise correlation (|*r*|>0.8) and reviewed variance inflation factor diagnostics. When multiple variables represented the same factor (eg, crude obesity prevalence and age-adjusted obesity prevalence), we retained a single variable to improve interpretability; furthermore, in this process, we prioritized the inclusion of SVI factors over AHRQ factors.^22^ This process resulted in the inclusion of six county-level sociodemographic factors from the AHRQ database: percentage of population who are veterans aged ≥18 years, children aged <18 years and living with grandparents, households with no computing device, households with a smartphone, households with broadband access, and households lacking complete kitchen facilities.

The final analytic predictor set comprised 34 factors: 18 SVI-based factors, 10 additional county-level factors from other public sources, and 6 AHRQ factors selected from ∼600 candidates using correlation screening (|*r*|>0.8), variance inflation factor review, and a one-variable-per-factor rule (final list of variables is provided in Supplementary Table 3).

### Data Preprocessing

For preprocessing, data were scaled using sci-kit-learn’s MinMaxScaler in order to equalize relative values of each feature.^23^ Then the data were randomly split into training (70%) and testing (30%) subsets using sci-kit-learn’s TrainTestSplit tool.^23^

### Defining Higher-Burden Counties

To leverage the predictive potential of binary classification models such as eXtreme Gradient Boosting (XGBoost) and support vector machine (SVM) models, we defined higher-burden counties as those with incidence of diagnosed diabetes >12.6 per 1000 persons, calculated from the sum of the mean (10.3 per 1000 persons) and standard deviation (SD) (2.3 per 1000 persons) from the overall sample. Based on this criterion, 500 counties were classified as having higher-burden (incidence >12.6 per 1000 persons) and 2614 counties as having lower-burden (incidence ≤12.6 per 1000 persons).

### ML Models

We considered three ML models: (1) elastic net regression, which provides insight from a regression model, with more interpretable feature coefficients; (2) SVM, which classifies counties with a higher-burden of incidence of diagnosed diabetes and provides detailed feature importance; and (3) XGBoost, which is a commonly used classification model for similar multivariable predictions such as stroke prevalence, health care costs, well-being of veterans, and improving model fairness for less represented populations.^24^, ^25^, ^26^, ^27^ XGBoost is less directly interpretable from model parameters alone; therefore, we used SHAP values to summarize feature effects on the model output.

#### Elastic Net Regression

Elastic net regression provides insight from a linear regression-based ML model. The benefit of elastic net regression over typical linear regression is the incorporation of lasso technique that allows for automatic feature selection during model training.^28^ This creates reproducible, consistent, and interpretable coefficients for key predictors for counties with a higher-burden of incidence of diagnosed diabetes. A grid search was conducted for hyperparameter fine-tuning on the training set (alpha = 0.001, l1_ratio = 0.9).

#### Support Vector Machine

A linear SVM model provides predictions based on a hyperplane created by the training data.^29^ This provides an interpretable and accurate predictions when used in a binary classification system. Feature importance provided by this method is consistent and explainable. Additionally, the binary predictions can be further examined on a percentage confidence basis. A grid search was conducted for hyperparameter fine-tuning, multiple kernels were evaluated, and a linear kernel (*C* = 1.0) was selected for interpretability.

#### eXtreme Gradient Boosting

XGBoost provides predictive insight from an ML model based on a tree boosting system. It offers general improvement in predictive scores, as well as computational resources required to run when compared to other tree boosting ML models.^30^ A grid search was conducted for hyperparameter fine-tuning: n_estimators = 200, max_depth = 4, learning_rate = 0.1, subsample = 0.8, colsample_bytree = 0.8, reg_lambda = 1.0.

### Model Verification

We used stratified fivefold cross-validation for classification models and fivefold cross-validation for regression.^23^ With each iteration, the model was trained on a different sampling of training vs testing data. Benchmarking scores, such as accuracy, precision, and recall, from each iteration were inspected to ensure consistency and reproducibility of the results. Sensitivity analyses were conducted for alternative definitions of higher-burden counties (Supplementary Table 4). Spatial autocorrelation was assessed to address potential spatial dependence that would influence model independence (Supplementary Table 5). Confusion matrices for SVM and XGBoost are shown in Supplementary Tables 6A and 6B. Given that ML-based feature importance measures are conditional on the trained model and lack standardized methods for uncertainty estimation, we did not report CIs for feature importance. Modeling was completed using Python 3.12.3 and supporting packages including JupyterLab 4.0.11, matplotlib 3.9.0, pandas 2.2.2, scikit-learn 1.5.0, and XGBoost 2.1.0.

## Results

In the overall sample of 3114 US counties, the mean (±SD) incidence of diagnosed diabetes was 10.3 ± 2.3 per 1000 persons, with an incidence of 14.0 ± 1.2 per 1000 persons in higher-burden counties (*N* = 500) and 9.6 ± 1.7 per 1000 persons in lower-burden counties (*N* = 2614) (*P* < .0001). Many sociodemographic factors differed between higher-burden and lower-burden counties (Table 1). Compared with lower-burden counties, higher-burden counties had lower per capita income, higher proportion of minority populations, children living with grandparent householders, obesity, and physical inactivity. In contrast, higher-burden counties had a lower proportions of adults with some college education and households with broadband access, with no significant difference in unemployment or availability of primary care physicians and other primary care professionals.

**Figure 1:**
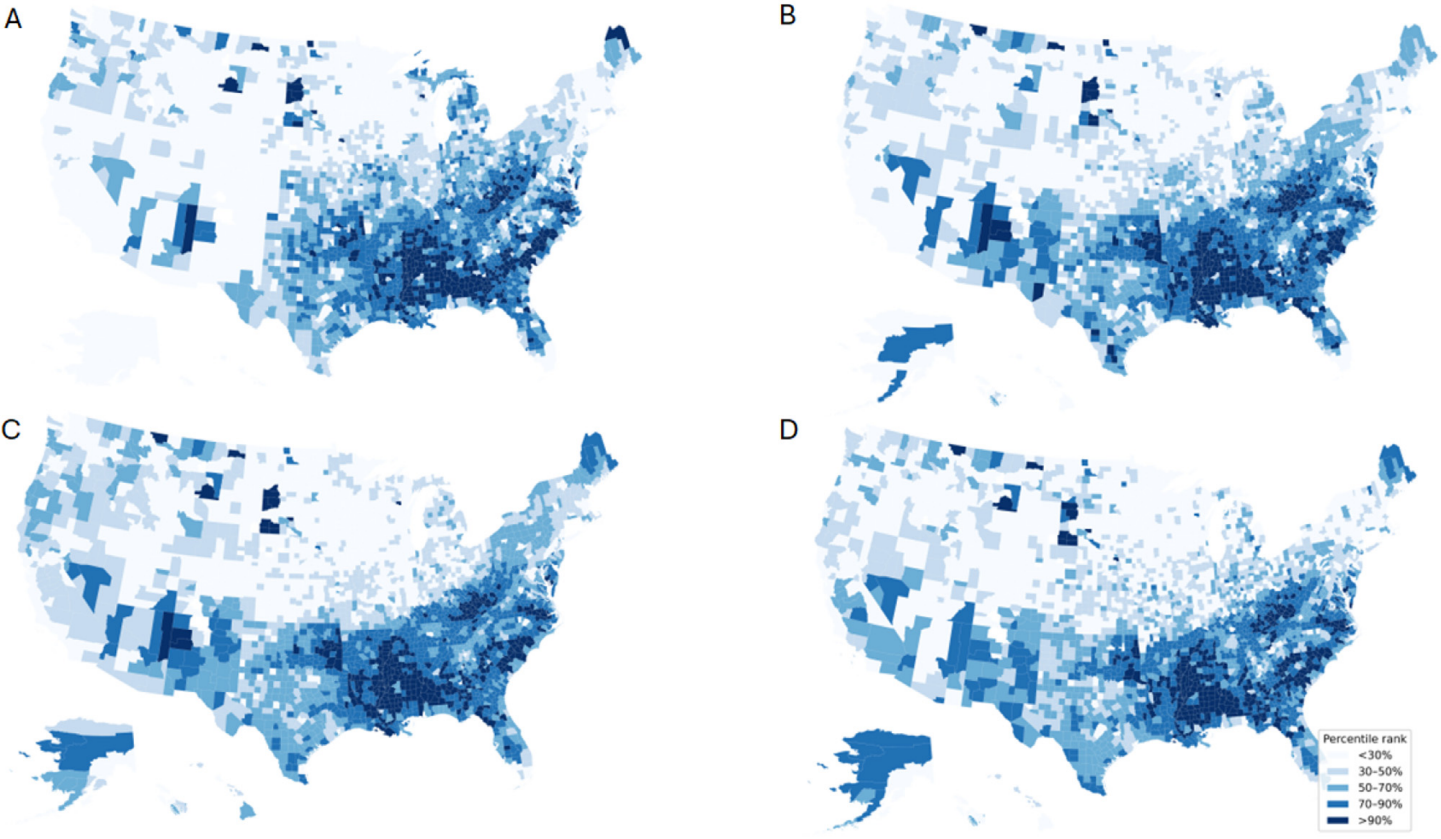
CDC and machine learning estimates of having a higher-burden of county-level incidence of diagnosed diabetes. Counties were categorized as having a higher-burden (incidence >12.6 per 1000 persons) or lower-burden (incidence ≤12.6 per 1000 persons) of incidence of diagnosed diabetes. The county-level burden of incidence is depicted based on (A) CDC estimates, (B) elastic net regression modeling, (C) support vector machine (SVM) modeling, and (D) XGBoost modeling. Elastic net regression predicted diabetes incidence. For SVM and XGBoost modeling, predicted higher-burden confidence was used, and lower-burden predictions were converted to a negative value. These ranges of values were binned by percentage to allow for visual comparison between each panel. Counties are depicted based on the likelihood of having a higher-burden, ranging from bottom 30% to top 10%.

Of the three ML models, XGBoost and SVM had high classification performance, with accuracies of 0.923 (95% CI, 0.905-0.939) and 0.924 (95% CI, 0.906-0.940), respectively. Elastic net regression had a mean absolute error of 0.810 (95% CI, 0.767-0.852) (Table 2). The XGBoost and SVM models demonstrated adequate and comparable precision, recall, and AUROC, with AUROC values of 0.957 (95% CI, 0.941-0.971) and 0.962 (95% CI, 0.948-0.974), respectively (Table 2). The confusion matrices for XGBoost and SVM are described in Supplementary Tables 6 and 7. Figure 1 displays each ML models predictions of diabetes incidence by US county, grouped by percentage range. All three models predicted that counties within the southern US region that had the highest diabetes incidence.

As counties in the same region may exhibit spatial dependence, we assessed residual spatial autocorrelation using Moran’s I with a region-based weights matrix (counties within the same census region were treated as neighbors). Diabetes incidence showed strong regional clustering (Moran’s *I* = 0.721; *P* = .001), whereas spatial autocorrelation of cross-validated residuals was lower for elastic net regression (*I* = 0.375; *P* = .001) and minimal or not statistically significant for SVM and XGBoost models (Supplementary Table 5).

Based on CDC data, we ranked US counties by their burden of incidence of diagnosed diabetes, with Greene County, Alabama, having the highest incidence (19.55 per 1000 persons) (Table 3). The top 25 counties were from seven US states: Alabama (8 counties), Mississippi (6 counties), Georgia (3 counties), South Dakota (3 counties), West Virginia (3 counties), Kentucky (1 county), and South Carolina (1 county). Of the three models, XGBoost had the high confidence in classifying most of the top 25 counties as having a higher-burden of incidence of diagnosed diabetes, followed by SVM. Elastic net regression predicted a lower incidence of diagnosed diabetes for all 25 counties, with the difference ranging from −4.04 per 1000 persons (Bullock, AL) to −0.54 per 1000 persons (Coahoma, MS).

**Table 3:** Counties Ranked by CDC Estimate of Incidence of Diagnosed Diabetes

County, State	CDC	Elastic Net Regression	XGBoost	SVM
	Incidence of Diagnosed Diabetes Per 1000 Persons	Incidence of Diagnosed Diabetes Per 1000 Persons; (Difference from CDC Estimate)	Confidence (%) in Predicting County as Higher-Burden	Confidence (%) in Predicting County as Higher-Burden
Greene, AL	19.55	15.81 (−3.74)	99.94	100.00
Lowndes, AL	19.00	16.18 (−2.82)	99.98	100.00
Perry, AL	18.40	16.42 (−1.98)	99.97	100.00
Bullock, AL	18.40	14.36 (−4.04)	98.66	94.30
Wilcox, AL	18.20	17.18 (−1.02)	99.99	100.00
Logan, WV	17.50	17.93 (+0.43)	99.89	100.00
Holmes, MS	17.50	14.44 (−3.06)	99.35	95.06
McDowell, WV	17.50	15.36 (−2.14)	97.68	99.13
Buffalo, SD	17.45	13.71 (−3.74)	98.57	93.01
Letcher, KY	17.35	14.28 (−3.07)	99.41	97.57
Boone, WV	17.20	13.55 (−3.65)	98.22	78.88
Sumter, AL	17.15	14.91 (−2.24)	99.98	99.59
Marengo, AL	17.10	15.94 (−1.16)	99.93	100.00
Jefferson, MS	17.10	15.47 (−1.63)	100.00	100.00
Kemper, MS	17.00	15.40 (−1.60)	99.91	99.23
Coahoma, MS	16.95	16.41 (−0.54)	99.95	100.00
Fairfield, SC	16.90	14.75 (−2.15)	99.99	97.62
Jasper, MS	16.85	15.95 (−0.90)	99.99	99.44
Macon, AL	16.80	15.01 (−1.79)	99.74	99.26
Ziebach, SD	16.65	14.30 (−2.35)	94.05	99.70
Dewey, SD	16.50	14.02 (−2.48)	99.45	97.93
Hancock, GA	16.45	13.39 (−3.06)	80.71	90.31
Talbot, GA	16.40	13.69 (−2.71)	99.90	90.97
Sharkey, MS	16.30	15.16 (−1.14)	99.63	99.43
Randolph, GA	16.25	15.73 (−0.52)	99.19	100.00

Counties were ranked based on the CDC estimated incidence of diagnosed diabetes. Also reported is the corresponding incidence of diagnosed diabetes predicted by elastic net regression and the confidence in being classified as a higher-burden county (incidence of diagnosed diabetes >12.6 per 1000 persons) by XGBoost and SVM modeling.

AL = Alabama; CDC = Centers for Disease Control and Prevention; GA = Georgia; KY = Kentucky; MS = Mississippi; SC = South Carolina; SD = South Dakota; SVM = support vector machine; WV = West Virginia; XGBoost = eXtreme Gradient Boosting.

**Figure 2:**
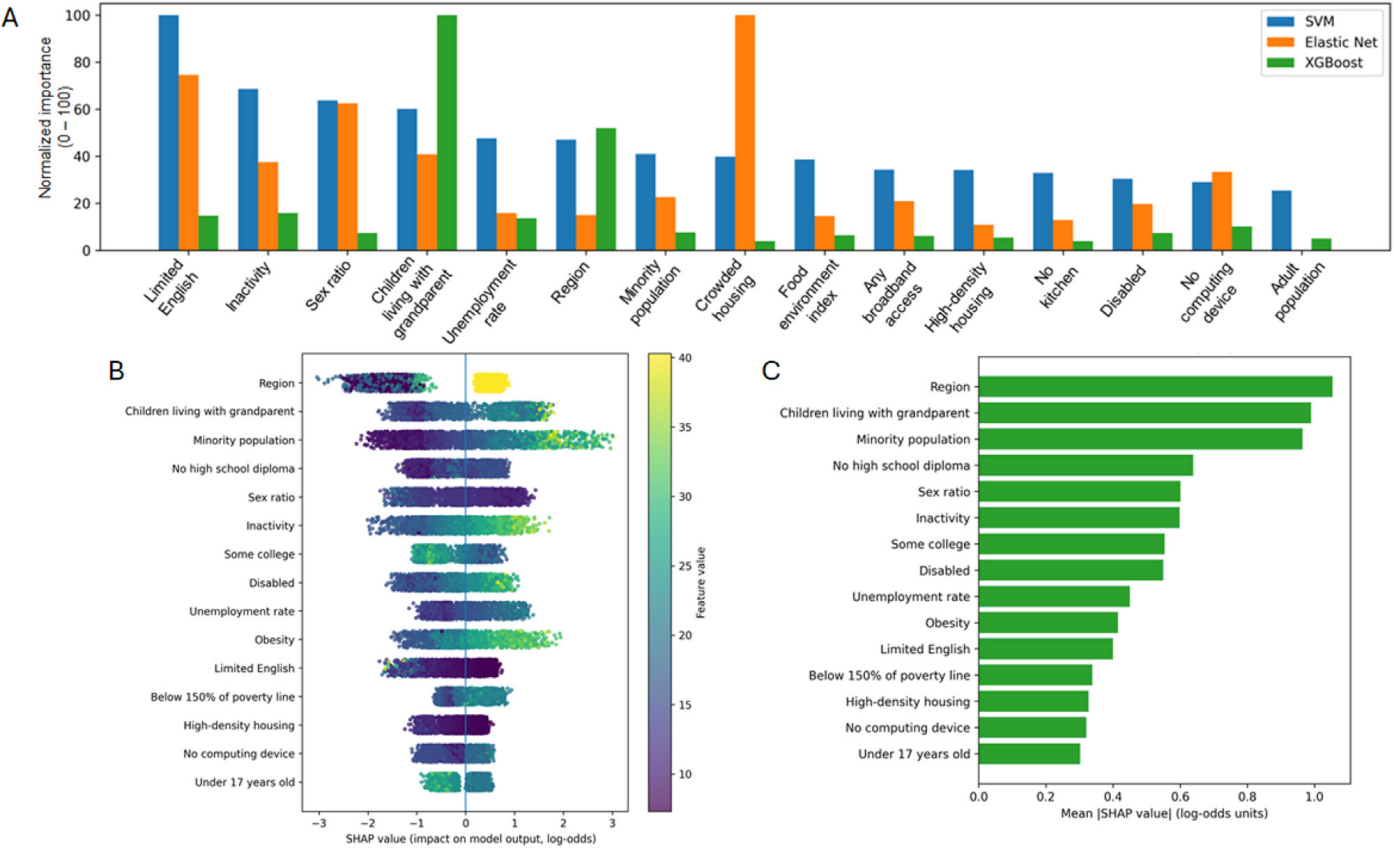
Feature importances by model. Top 15 sociodemographic features and incidence of diagnosed diabetes, sorted by feature importance from the SVM model. (A) Features are depicted in decreasing order of importance. Each feature has three bars, depicting results from SVM (blue), elastic net regression (orange), and XGBoost (green) modeling. (B) TreeSHAP summary (beeswarm) plot for the XGBoost model. The *x*-axis shows SHAP values, indicating the direction and magnitude of each feature’s contribution to incidence of diagnosed diabetes (model output). Positive SHAP values indicate contributions toward a higher predicted probability of the outcome, while negative values indicate contributions toward a lower predicted probability. Colors indicate the relative feature value (low to high). (C) Mean absolute SHAP value for each feature across all observations for the XGBoost model, reflecting the overall magnitude of each feature’s contribution to incidence of diagnosed diabetes (model output). Higher values indicate higher influence, but not directionality, on model predictions. The top 25 factors are listed in Supplementary Table 7.

Several sociodemographic factors had high feature importance for predicting counties with a higher-burden of incidence of diagnosed diabetes. Figure 2 shows the top-ranked features by feature importance across models. The highest-ranked features differed by model (Figure 2; Supplementary Table 7), and differences likely reflect model class (linear regularized vs nonlinear) and correlated predictors. We highlighted SVM coefficient-based feature importance for interpretability and used SHAP summaries to interpret the XGBoost model (Figure 2). The features with the highest importance were *Limited English* (SVM), *children living with grandparent householders* (XGBoost), and *crowded housing* (elastic net regression) (Figure 2). Across all three models, *children living with grandparent householders* and *crowded housing* were in the top 5 features (Supplementary Table 7). Other key features identified in all three models were inactivity, region, no computing device, whereas SVM and elastic net regression models additionally identified obesity, minority population, and single-parent household (Figure 2 and Supplementary Table 7). However, there were some differences in feature importance across the three models. These differences likely reflect methodological differences among modeling approaches: Elastic net regression prioritizes linear associations and performs feature selection through regularization, whereas SVM and XGBoost capture nonlinear relationships and higher-order interactions. In addition, the models apply different weights to correlated sociodemographic variables, which may result in different feature importance.

In the XGBoost model, the TreeSHAP summary (beeswarm) plot identified *region, children living with grandparent householders*, and *minority populations* as the most influential features contributing to predicted diabetes incidence, with heterogeneous effects across observations (Figure 2B). The SHAP global feature importance plot depicts the overall magnitude of each feature’s contribution to model predictions based on mean absolute SHAP values (Figure 2C).

### Additional Analysis

Higher-burden counties were defined based on incidence >12.64 per 1000 persons (mean + SD). In sensitivity analyses defining higher-burden counties as incidence >12.06 per 1000 persons (mean + 0.75 × SD) and incidence >13.21 per 1000 persons (mean + 1.25 × SD), model discrimination (AUROC) was similar, with expected changes in F1 score as the prevalence of higher-burden counties changed (Supplementary Table 4).

## Discussion

In this US study, 500 of 3114 counties were defined as having a higher-burden of incidence of diagnosed diabetes. Higher-burden and lower-burden counties differed in 21 of 34 sociodemographic factors evaluated. Notably, higher-burden counties had a higher percentage of *children living with grandparent householders*, obesity, and physical inactivity, but lower prevalence of technology-related factors such as broadband access and smartphones. Across all three ML models, *children living with grandparent householders* and *Limited English* were important features contributing to model predictions of diabetes incidence (elastic net regression) and classification of higher-burden counties (SVM, XGBoost).

To our knowledge, *children living with grandparent householders* has not been evaluated as a county-level factor contributing to predicted diabetes incidence. An analysis of the US National Survey of Children’s Health data, from 2017 to 2018, reported that children (aged 10-17 years) living in grandparent-headed households had a high burden of adverse childhood experiences that were independently associated with poor cardiometabolic indicators such as obesity and physical activity.^31^ Children may live with grandparents in co-resident households (ie, parents also residing) and skip-generation households (ie, parents not residing), with the latter potentially reflecting socioeconomic disadvantage and parental challenges with employment, physical and mental health, and/or incarceration.^32^, ^33^, ^34^ Although these studies focused on individual- or household-level outcomes, our findings suggest that the prevalence of grandparent households may serve as a county-level marker of socioeconomic stressors relevant to diabetes burden. In future work, we will stratify counties by their percentages of *children living with grandparent householders* to gain insight into sociodemographic profiles associated with diabetes incidence.

The positive association of obesity and inactivity, and inverse association of food environment index, with incidence of diagnosed diabetes builds on previous work from us and others.^2^^,^^9^ US county-level analysis showed that counties with exercise opportunities had 15.7 per 100,000 persons lower incident cases whereas 1 unit increment in food environment index (ie, indicating better environment) was associated with 19.7 per 100,000 persons lower incident cases.^9^ In the present study, SVM modeling showed that *crowded housing* and *no kitchen* had high feature importance, which in the setting of high *employment rate* and low *food environment index* may compound barriers to healthy food access. A recent 6-week cooking intervention showed improvement diabetes self-management outcomes.^35^ Taken together with growing evidence that certain diets (eg, Mediterranean diet) reduce risk of incident diabetes, future studies should evaluate if culturally tailored, affordable cooking can be sustained to prevent diabetes.^36^^,^^37^

The present study identified technology-related factors (ie, no computing device, smartphone, broadband access) as having high feature importance, which may limit access to health information, self-assessment scores for current risk of diabetes, technology-dependent health interventions, and primary prevention clinical trials. These findings suggest that technology-dependent interventions may have limited reach in counties with lower technology access, underscoring the importance of aligning prevention strategies with local infrastructure.^38^, ^39^, ^40^ From a public health perspective, these findings highlight how ML–based approaches can identify county-level contexts in which combinations of sociodemographic constraints may influence diabetes prevention strategies, underscoring the need for geographically tailored interventions.

In the present study, all three ML models identified region as an important feature. Previously, we reported that the US South, compared with other US regions, had the highest incidence of diagnosed diabetes.^2^ Although not evaluated in the present study, we have previously reported regional differences in prevalence of sociodemographic factors.^2^ For example, at the county-level, the US South has lower median income, higher proportion of uninsured persons, and a lower proportion of primary care physicians, which may influence risk of incident diabetes.^2^^,^^41^^,^^42^ In SVM and elastic net regression models, county-level rurality was not identified in the top 10 of feature importance; this is consistent with our previous finding that *rurality* likely does not confer an independent risk for diabetes incidence after accounting for sociodemographic factors, as demonstrated in the present study.

We used ML approaches, which have been increasingly used to predict outcomes related to chronic diseases such as diabetes and cardiovascular disease.^43^^,^^44^ ML offers greater flexibility with fewer *a priori* assumptions, identifies robust predictors and interactions across multiple models, and often achieves higher predictive performance than traditional regression in large-scale, high-dimensional data.^45^^,^^46^ ML approaches can identify important factors across various model types (eg, SVM, XGBoost, Elastic Net), and cross-model consensus increases confidence in interpreting the associations between sociodemographic features and outcomes. Furthermore, ML models are computationally efficient and scalable, enabling detailed analysis of large population-based datasets.^46^ In the present study, the three ML models served complementary purposes to estimate diabetes incidence (elastic net regression) or classify counties as having a higher-burden of diabetes (SVM, XGBoost). Although there was some overlap in feature importance across the three models, these findings should be interpreted as hypothesis-generating and not causal.

There are limitations to this study, as previously reported.^2^ The analysis was performed at the county-level, and the findings may not apply to individuals. The CDC used self-reported physical inactivity and height and weight (to calculate body mass index and define obesity), which are subject to reporting and recall bias. However, the use of multi-year, county-level aggregated data may have reduced random measurement error. The CDC used self-reported diabetes, which included both type 1 and type 2 diabetes. However, in adults, 90% to 95% of diabetes cases are due to type 2 diabetes, and our findings likely pertain to type 2 diabetes. We included a broad set of sociodemographic factors; however, residual confounding by unmeasured or incompletely measured county-level factors is possible. County-level measures were not statistically independent, as neighboring counties share geographic, social, and health system characteristics. While this nonindependence affects inference in traditional regression models, the ML approaches used in the present study focused on predictive performance rather than parameter estimation. Although we used three ML approaches, these methods only evaluated associations and do not imply a causal or mechanistic relationship between sociodemographic factors and diabetes. The study has several strengths. We aggregated data over a 16-year period, from 2004 to 2019, as previously reported, and to yield more stable estimates of incidence.^2^ Temporal aggregation prioritizes spatial patterning and relative burden across counties but does not capture within-county temporal trends or changes over time. We considered more than 600 sociodemographic factors from public databases, which, to our knowledge, is the most comprehensive evaluation. Finally, as this study evaluated county-level sociodemographic factors and incidence of diagnosed diabetes, the findings may be more applicable to tailored, population-level interventions, which may reduce diabetes incidence and other cardiometabolic conditions with overlapping risk factors.

## Conclusion

This study identified county-level sociodemographic factors contributing to ML model-based estimates of diabetes incidence and classification of higher-burden counties. The identification of established, emerging, and less commonly evaluated factors may inform future efforts to characterize geographic patterns of diabetes incidence and guide population-level prevention strategies.

## Declaration of Generative AI and AI-Assisted Technologies in the Writing Process

During the preparation of this work, the authors used ChatGPT (within the Mayo Clinic secure cloud environment) in order to edit text. After using this large language model, the authors reviewed and edited the content as needed and take full responsibility for the content of the published article.

## CRediT authorship contribution statement

**Alexander S. Keigley:** Writing – review & editing, Writing – original draft, Visualization, Validation, Software, Methodology, Investigation, Formal analysis, Conceptualization. **Shant Ayanian:** Writing – review & editing, Writing – original draft, Validation, Supervision, Methodology, Funding acquisition, Formal analysis, Data curation, Conceptualization. **Sagar B. Dugani:** Writing – review & editing, Supervision, Resources, Project administration, Methodology, Investigation, Funding acquisition, Conceptualization.

## Declaration of competing interest

S.B.D. was supported by the National Institutes of Health/National Institute on Minority Health and Health Disparities (NIH K23 MD016230) and Mayo Clinic Center for Clinical and Translational Science (UL1 TR02377-07). Other authors declare that they have no known competing financial interests or personal relationships that could have appeared to influence the work reported in this article. The funders had no role in study design, data analysis and interpretation; in writing of the manuscript; and in the decision to submit the manuscript for publication. The findings and conclusions do not necessarily represent the views of the funders.

## Appendix Supplementary materials

Image, application 1
