# Supplementary material for: Machine Learning Models to Evaluate County-Level Incidence of Diagnosed Diabetes and Sociodemographic Factors
Source: Am J Med Open. 2026 Mar 27;15:100132. doi: 10.1016/j.ajmo.2026.100132 (PMC13141798; doi:10.1016/j.ajmo.2026.100132)
Supplement: Supplementary file 1 [file mmc1.docx]

**SUPPLEMENTARY ONLINE CONTENT**

**Machine Learning Models to Evaluate County-level Incidence of Diagnosed Diabetes and Sociodemographic Factors**

AS Keigley, S Ayanian, SB Dugani

| **Name** | **Title** |
| --- | --- |
| Supplementary Table 1 | County-level rurality definition |
| Supplementary Table 2 | US regions and states |
| Supplementary Table 3 | County-level sociodemographic factors |
| Supplementary Table 4 | Sensitivity analysis of different thresholds to define higher-burden counties |
| Supplementary Table 5 | Spatial autocorrelation in observed incidence and cross-validated model residuals |
| Supplementary Table 6 | Confusion matrixes for XGBoost and SVM |
| Supplementary Table 7 | Machine learning feature importance and county-level incidence of diagnosed diabetes |

**Supplementary Table 1: County-level rurality definition**

| **County rurality** | **Metropolitan status** | **Definition** |
| --- | --- | --- |
| Large central metro | metropolitan | Counties in MSAs of 1 million or more population that:  1. Contain the entire population of the largest principal city of the MSA, or  2. Have their entire population contained in the largest principal city of the MSA, or  3. Contain at least 250,000 inhabitants of any principal city of the MSA. |
| Large fringe metro | metropolitan | Counties in MSAs of 1 million or more population that did not qualify as large central metro counties. |
| Medium metro | metropolitan | Counties in MSAs with population 250,000─999,999. |
| Small metro | metropolitan | Counties in MSAs with population less than 250,000. |
| Micropolitan | nonmetropolitan | Counties in micropolitan statistical areas. |
| Noncore | nonmetropolitan | Nonmetropolitan counties that did not qualify as micropolitan. |

Rurality definitions were from the CDC 2013 NCHS Urban–Rural Classification Scheme for Counties^19^.

Abbreviation: MSA metropolitan statistical area.

       From Dugani et al., with permission.**^2^**

**Supplementary Table 2: US regions and states**

| **Regions** | **States** |
| --- | --- |
| Northeast | Connecticut; Maine; Massachusetts; New Hampshire; New Jersey; New York; Pennsylvania; Rhode Island; Vermont |
| Midwest | Indiana; Illinois; Iowa; Kansas; Michigan; Minnesota; Missouri; Nebraska; North Dakota; Ohio; South Dakota; Wisconsin |
| South | Alabama; Arkansas; Delaware; District of Columbia; Florida; Georgia; Kentucky; Louisiana; Maryland; Mississippi; North Carolina; Oklahoma; South Carolina; Tennessee; Texas; Virginia; West Virginia |
| West | Alaska; Arizona; California; Colorado; Hawaii; Idaho; Montana; New Mexico; Oregon; Utah; Nevada; Washington; Wyoming |

       From Dugani et al., with permission.^2^

**Supplementary Table 3: County-level sociodemographic factors**

| Sociodemographic Factors | Definition | Source |
| --- | --- | --- |
| Adult population^47^ | Persons aged ≥20 years | US Census ACS, 2009, 2014, and 2019 (5-year estimates). |
| Over 65 years old^17^ | Percentage of persons aged ≥65 years | US Census ACS, 2014, 2016, 2018, 2020, and 2022 (5-year estimates). |
| Under 17 years old^17^ | Percentage of persons aged ≥17 years | US Census ACS, 2014, 2016, 2018, 2020, and 2022 (5-year estimates). |
| Sex ratio^47^ | Ratio of men to women | US Census ACS, 2009, 2014, and 2019 (5-year estimates). |
| No high school diploma^17^ | Percentage of persons with no high school diploma (25+) | US Census ACS, 2014, 2016, 2018, 2020, and 2022 (5-year estimates). |
| Some college^20^ | Proportion of population aged 25-44 years with some post-secondary education, such as enrollment in vocational/technical schools, junior colleges, or 4-year colleges. It includes individuals who pursued education following high school but did not receive a degree as well as those who attained degrees. | US Census ACS, 2011 (years 2005–2009), 2016 (years 2010–2014),  and 2021 (years 2015–2019) (5-year estimates). |
| Per capita income^17^ | Per capita income | US Census ACS, 2014, 2016, 2018, 2020, and 2022 (5-year estimates). |
| Unemployment rate^17^ | Unemployment rate | US Census ACS, 2014, 2016, 2018, 2020, and 2022 (5-year estimates). |
| Below poverty line^17^ | Percentage of persons below poverty | US Census ACS, 2014, 2016, 2018, 2020, and 2022 (5-year estimates). |
| Below 150% of poverty line^17^ | Percentage of persons below 150% of poverty line | US Census ACS, 2014, 2016, 2018, 2020, and 2022 (5-year estimates). |
| Uninsured^17^ | Percentage uninsured in the total civilian noninstitutionalized population | US Census ACS, 2014, 2016, 2018, 2020, and 2022 (5-year estimates). |
| Food environment index^20^ | Food Environment Index equally weights two indicators of the food environment:   - Limited access to healthy foods estimates the percentage of the population that is low income and does not live close to a grocery store. Low income is defined as having an annual family income ≤200% of federal poverty threshold for the family size. Living close to a grocery store is defined differently in rural and nonrural areas; <10 miles (rural) and <1 mile (nonrural). - Food insecurity estimates the percentage of the population that did not have access to a reliable source of food during the past year.   Food environment index was measured on a scale from 0 to 10, with higher values representing increased access to healthy food choices. | USDA Food Environment Atlas, Map the Meal Gap, 2010–2011, 2012, 2013, 2014.  USDA Food Environment Atlas, Map the Meal Gap from Feeding America, 2015-2016, 2019. |
| Veterans^18^ | Percentage of civilian population consisting of veterans (aged ≥18 years) | US Census ACS 2009-2020 (5-year estimates). |
| Minority population^17^ | Percentage minority (Hispanic or Latino (of any race); Black and African American, Not Hispanic or Latino; American Indian and Alaska Native, Not Hispanic or Latino; Asian, Not Hispanic or Latino; Native Hawaiian and Other Pacific Islander, Not Hispanic or Latino; Two or More Races, Not Hispanic or Latino; Other Races, Not Hispanic or Latino) estimate | US Census ACS, 2014, 2016, 2018, 2020, and 2022 (5-year estimates). |
| Disabled^17^ | Percentage of civilian noninstitutionalized population with a disability | US Census ACS, 2014, 2016, 2018, 2020, and 2022 (5-year estimates). |
| Limited English^17^ | Percentage of persons (age 5+) who speak English “less than well” | US Census ACS, 2014, 2016, 2018, 2020, and 2022 (5-year estimates). |
| Single parent households^17^ | Percentage of single-parent households with children aged <18 years | US Census ACS, 2014, 2016, 2018, 2020, and 2022 (5-year estimates). |
| Children living with grandparent householders^18^ | Percentage of children living with a grandparent householder (aged ≤17 years). This includes households both with and without parents. | US Census ACS 2009-2020 (5-year estimates). |
| Mobile homes^17^ | Percentage of mobile homes | US Census ACS, 2014, 2016, 2018, 2020, and 2022 (5-year estimates). |
| High-density housing^17^ | Percentage of housing in structures with 10 or more units | US Census ACS, 2014, 2016, 2018, 2020, and 2022 (5-year estimates). |
| Crowded housing^17^ | Percentage of occupied housing units with more people than rooms | US Census ACS, 2014, 2016, 2018, 2020, and 2022 (5-year estimates). |
| Group quarters^17^ | Percentage of persons in group quarters | US Census ACS, 2014, 2016, 2018, 2020, and 2022 (5-year estimates). |
| Housing cost-burden^17^ | Percentage of housing cost-burdened occupied housing units with annual income less than $75,000 (30% of income spent on housing costs) | US Census ACS, 2014, 2016, 2018, 2020, and 2022 (5-year estimates). |
| No vehicle available^17^ | Percentage of households with no vehicle available | US Census ACS, 2014, 2016, 2018, 2020, and 2022 (5-year estimates). |
| No computing device^18^ | Percentage of households without a computing device | US Census ACS 2017-2020 (5-year estimates). |
| Smartphone^18^ | Percentage of households with a smartphone | US Census ACS 2017-2020 (5-year estimates). |
| Any broadband access^18^ | Percentage of households with broadband of any type | US Census ACS 2017-2020 (5-year estimates). |
| No kitchen^18^ | Percentage of housing units lacking complete kitchen facilities | US Census ACS 2009-2020 (5-year estimates). |
| Primary care physicians^20^ | Ratio of primary care physicians to county population. Primary care physicians include practicing non-federal physicians (MDs and DOs) under age 75 years specializing in general practice medicine, family medicine, internal medicine, and pediatrics. | HRSA Area Resource File, 2011.  Area Health Resource File/American Medical Association, 2012, 2013, 2014, 2015, 2016, 2017, 2018, 2019. |
| Other primary care professionals^20^ | Ratio of primary care providers other than physicians to county population. | CMS, National Provider Identification, 2014, 2015, 2016, 2017, 2018, 2019. |
| Obesity^47^ | Prevalence of obesity, % | Obesity was defined as body-mass index of ≥30 kg/m^2^, which was derived from self-reported height and weight during the BRFSS interview.  CDC Diabetes County Data Indicators, 2006–2017. |
| Inactivity^47^ | Prevalence of leisure-time physical inactivity, % | Leisure-time physical inactivity (yes/no) was based on self-reported “no” to the question during the BRFSS interview: "During the past month, other than your regular job, did you participate in any physical activities or exercises such as running, calisthenics, golf, gardening, or walking for exercise?"  CDC Diabetes County Data Indicators, 2006–2017. |
| County-level rurality^19^ | Categorical measure of rurality. See Supplementary Table 1. | CDC 2013 NCHS Urban–Rural Classification Scheme for Counties. |
| Region | Geographical area of the United States. See Supplementary Table 2. | US Census Bureau classification: Northeast, Midwest, South, and West. |

Sociodemographic factors were extracted from Rural Health Information Hub^47^, NCHS urban-rural classification scheme for counties^19^, County Health Rankings & Roadmaps^20^, Center for Disease Control with Agency for Toxic Substances and Disease Registry Social Vulnerability Index^17^, and Agency for Healthcare Research and Quality Social Determinants of Health Database^18^.

Abbreviations: ACS American Community Survey; CDC Centers for Disease Control and Prevention; CMS Centers for Medicare & Medicaid Services; DO Doctor of Osteopathy; MD Doctor of Medicine; NCHS National Center for Health Statistics; US United States; USDA US Department of Agriculture

**Supplementary Table 4: Sensitivity analysis of different thresholds to define higher-burden counties**

| **Burden of diabetes incidence** | | | | **SVM** | | | | | **XGBoost** | | | | |
| --- | --- | --- | --- | --- | --- | --- | --- | --- | --- | --- | --- | --- | --- |
| **Cutoff threshold** | **Incidence cutoff** | **No of counties** | | **AUROC** | **Accuracy** | **Precision** | **Recall** | **F1 score** | **AUROC** | **Accuracy** | **Precision** | **Recall** | **F1**  **score** |
|  |  | **Higher-burden** | **Lower-burden** |  |  |  |  |  |  |  |  |  |  |
| Mean + 0.75×SD | 12.06 | 681 | 2433 | 0.960 | 0.908 | 0.807 | 0.760 | 0.783 | 0.961 | 0.917 | 0.842 | 0.760 | 0.799 |
| Mean + 1 SD | 12.64 | 500 | 2614 | 0.962 | 0.924 | 0.821 | 0.673 | 0.740 | 0.957 | 0.923 | 0.800 | 0.693 | 0.743 |
| Mean + 1.25×SD | 13.21 | 343 | 2771 | 0.969 | 0.936 | 0.765 | 0.602 | 0.674 | 0.967 | 0.940 | 0.747 | 0.689 | 0.717 |

Sensitivity analysis of different thresholds to define higher-burden and lower-burden counties. In the main analysis, ‘mean + 1 SD’ was used to define the threshold. Sensitivity analysis conducted at different thresholds: ‘mean + 0.75 SD’ and ‘mean + 1.25 SD’. Incidence cut-off is per 1000 persons.

Abbreviations: AUROC: area under receiver operative curve; SD: standard deviation; SVM: support vector machine; XGBoost: eXtreme Gradient Boosting

**Supplementary Table 5: Spatial autocorrelation in observed incidence and cross-validated model residuals**

| **Measure** | **Moran's I** | **Permutation *P* value** |
| --- | --- | --- |
| Observed incidence | 0.721 | 0.001 |
| Elastic net regression residuals | 0.375 | 0.001 |
| SVM residuals | 0.04 | 0.001 |
| XGBoost residuals | -0.002 | 0.874 |

Spatial autocorrelation was assessed using Moran’s I applied to 5-fold out-of-fold residuals. A region-based spatial weights matrix was used, with counties within the same U.S. Census region (Northeast, South, Midwest, West) treated as neighbors , and statistical significance evaluated using 999 permutations.

Abbreviations: SVM: support vector machine; XGBoost: eXtreme Gradient Boosting

**Supplementary Table 6A: Confusion matrix for XGBoost**

|  |  | Predicted burden, from XGBoost | |
| --- | --- | --- | --- |
|  |  | Lower-burden | Higher-burden |
| True burden, from CDC | Lower-burden | 759 | 26 |
|  | Higher-burden | 46 | 104 |

Counties were categorized based on incidence of diagnosed diabetes per 1000 persons:

higher-burden (>12.6) and lower-burden (≤12.6), based on mean incidence + 1 SD cutoff.

True burden based on CDC report and predicted burden based on XGBoost. The confusion matrix

is based on a 30% held-out test set.

Abbreviations: CDC: Centers for Disease Control and Prevention; XGBoost: eXtreme Gradient Boosting

**Supplementary Table 6B: Confusion matrix for SVM**

|  |  | Predicted burden, from SVM | |
| --- | --- | --- | --- |
|  |  | Lower-burden | Higher-burden |
| True burden, from CDC | Lower-burden | 763 | 22 |
|  | Higher-burden | 49 | 101 |

Counties were categorized based on incidence of diagnosed diabetes per 1000 persons:

higher-burden (>12.6) and lower-burden (≤12.6), based on mean incidence + 1 SD cutoff.

True burden based on CDC report and predicted burden based on SVM. The confusion matrix

is based on a 30% held-out test set.

Abbreviations: CDC: Centers for Disease Control and Prevention; SVM: support vector machine

**Supplementary Table 7: Machine learning feature importance and county-level incidence of diagnosed diabetes**

| Feature | SVM | Elastic Net Regression | XGBoost |
| --- | --- | --- | --- |
| Limited English | 100.0 | 74.59 | 14.76 |
| Inactivity | 68.68 | 37.56 | 15.85 |
| Sex ratio | 63.81 | 62.55 | 7.39 |
| Children living with grandparent householders | 60.14 | 40.83 | 100.0 |
| Unemployment rate | 47.71 | 15.9 | 13.59 |
| Region | 47.05 | 15.04 | 51.96 |
| Minority population | 40.99 | 22.66 | 7.64 |
| Crowded housing | 39.93 | 100.0 | 3.93 |
| Food environment index | 38.65 | 14.66 | 6.4 |
| Any broadband access | 34.34 | 21.04 | 6.15 |
| High-density housing | 34.23 | 10.92 | 5.41 |
| No kitchen | 32.93 | 12.92 | 3.9 |
| Disabled | 30.4 | 19.69 | 7.38 |
| No computing device | 29.03 | 33.36 | 10.23 |
| Adult population | 25.42 | 0.0 | 5.1 |
| Group quarters | 24.31 | 0.0 | 5.18 |
| Some college | 24.31 | 32.43 | 4.93 |
| Obesity | 24.19 | 23.55 | 8.94 |
| Over 65 years old | 23.77 | 37.35 | 4.61 |
| Single parent households | 21.0 | 23.62 | 5.25 |
| Below poverty line | 20.82 | 1.22 | 3.81 |
| No vehicle available | 18.08 | 0.0 | 12.57 |
| Uninsured | 12.32 | 7.01 | 6.25 |
| Other primary care professionals | 12.2 | 0.0 | 4.12 |
| Under 17 years old | 9.62 | 0.0 | 3.76 |
| No high school diploma | 9.17 | 21.34 | 8.68 |
| Smartphone | 7.08 | 27.11 | 3.4 |
| Per capita income | 6.71 | 25.86 | 3.3 |
| Below 150% of poverty line | 4.13 | 0.76 | 7.84 |
| Veterans | 3.68 | 1.18 | 3.3 |
| County-level rurality | 3.51 | 5.68 | 1.93 |
| Mobile homes | 2.18 | 3.38 | 4.05 |
| Housing cost-burden | 0.68 | 47.97 | 2.85 |
| Primary care physicians | 0.35 | 15.19 | 3.6 |

Feature importances, sorted by SVM feature importances. Feature importance refers to the strength of a feature in predicting incidence of diagnosed diabetes (Elastic Net Regression) or if a county has a higher-burden of incidence of diagnosed diabetes (SVM and XGBoost).

Abbreviations: SVM: support vector machine; XGBoost: eXtreme Gradient Boosting
